# Renin-Angiotensin System Single Nucleotide Polymorphisms Are Associated with Bladder Cancer Risk

**DOI:** 10.3390/curroncol28060396

**Published:** 2021-11-15

**Authors:** Maria Samara, Maria Papathanassiou, Ioanna Farmakioti, Maria Anagnostou, Maria Satra, Lampros Mitrakas, Dimitrios Anastasiou, Georgios Chasiotis, Agamemnon Christopoulos, Athanasios Anagnostou, Anastasios Christodoulou, Alexandros Daponte, Maria Ioannou, George Koukoulis, Vassilios Tzortzis, Panagiotis J. Vlachostergios

**Affiliations:** 1 Department of Pathology, Faculty of Medicine, School of Health Sciences, University of Thessaly, 41100 Larissa, Greece; msamar@uth.gr (M.S.); mpapat@uth.gr (M.P.); ioannafarmakioti@gmail.com (I.F.); managnostou@bio.uth.gr (M.A.); mioan@uth.gr (M.I.); kougeo@uth.gr (G.K.); 2 Department of Biology, Faculty of Medicine, School of Health Sciences, University of Thessaly, University Hospital of Larissa, 41100 Larissa, Greece; msatra@uth.gr; 3 Department of Urology, Faculty of Medicine, School of Health Sciences, University of Thessaly, University Hospital of Larissa, 41100 Larissa, Greece; lmitrak@uth.gr (L.M.); jimious23@gmail.com (D.A.); geochas92@gmail.com (G.C.); christopoulosagamemnon@gmail.com (A.C.); nasos_85@hotmail.com (A.A.); tasoschristodoulou@windowslive.com (A.C.); 4 Department of Obstetrics and Gynecology, Faculty of Medicine, University of Thessaly, 41100 Larissa, Greece; daponte@uth.gr; 5 Department of Medicine, Division of Hematology and Medical Oncology, Weill Cornell Medicine, New York, NY 10065, USA

**Keywords:** renin-angiotensin system, angiotensinogen, bladder cancer, single nucleotide polymorphisms, angiogenesis

## Abstract

The renin-angiotensin system (RAS), besides being a major regulator of blood pressure, is also involved in tumor angiogenesis. Emerging evidence suggests a correlation between the use of pharmacologic RAS inhibitors and a delay in urothelial bladder cancer (BC) progression. However, it is unknown whether RAS gene variants may predispose to the development of BC. This study examined the association of RAS single nucleotide polymorphisms (SNPs) including AT1R rs5186, AT2R rs11091046, REN rs12750834, ANG rs4762, and ANG rs699 with the risk of developing non-invasive BC. Peripheral blood samples from 73 patients with T1 urothelial BC (66 men, seven women) and an equal number of healthy subjects (control group) were collected. The TT genotype of the REN rs12750834 SNP (OR: 2.8 [1.3–6.05], *p* = 0.008) and to a lesser extent the presence of the T allele (OR: 2.3 [1.2–4.48], *p* = 0.01) conferred a higher risk of BC. The highest risk for BC within SNP carriers of the RAS system was associated with the presence of the CC genotype (OR: 17.6 [7.5–41.35], *p* < 0.001) and C allele (OR: 17.7 [8.8–35.9], *p* < 0.001) of the ANG rs699 SNP. The presence of the AT2R rs11091046 SNP, particularly the AA genotype, was associated with a protective effect against developing BC (OR: 0.268 [0.126–057], *p* < 0.001). In conclusion, these results support the clinical utility of RAS gene SNPs AT2R rs11091046, REN rs12750834, and ANG rs699 in the genetic cancer risk assessment of patients and families with BC.

## 1. Introduction

Urothelial carcinogenesis is a multistep process involving two major divergent pathways with distinct molecular and clinical characteristics, non-invasive and muscle-invasive disease [1,2,3]. The unraveling of the underlying biology of these entities has provided a wealth of targets within distinct molecular pathways [4]. One key aspect of bladder cancer (BC) is angiogenesis. The most well-studied and clinically relevant angiogenic mediator in both invasive and non-invasive BC is vascular endothelial growth factor (VEGF), with a high expression being associated with deeper, high-grade, aggressive tumors of the bladder [5] and elevated urinary VEGF levels predicting recurrence of superficial BC [6]. However, the role of other angiogenic players, including members of the renin angiotensin system (RAS) is often underscored.

Key components of RAS, such as angiotensin and its receptors, type 1 (AT1R) and type 2 (AT2R), demonstrate an inverse expression pattern in BC, involving AT1R upregulation and AT2R downregulation in human BC tumors [7]. Mechanistically, induced overexpression of AT2R results in caspase-dependent apoptosis in BC cells and regression of bladder tumor xenografts [7].

Pharmacologic inhibition of the renin-angiotensin system (RAS) with the use of angiotensin-converting enzyme inhibitors (ACEI) or angiotensin receptor blockers (ARBs) in patients with non-muscle-invasive BC can prolong the 3-year and 5-year recurrence-free survival, independently of other known prognostic factors, including tumor multiplicity and the absence of bacillus Calmette–Guérin instillation [8,9,10]. Renin-angiotensin system inhibitors have a similar effect, lowering the risk of cancer-specific and overall mortality after radical cystectomy [11]. While these findings support the clinical relevance of assessing the expression of the RAS system to predict disease progression, it is also true that subjects with common RAS gene variants, also known as single nucleotide polymorphisms (SNPs), may experience a different pharmacologic effect from these drugs [12,13,14].

Currently, it is poorly understood whether there is a relationship between RAS SNPs and the predisposition to BC. We hypothesized that RAS gene SNPs that were associated with hypertension and/or decreased efficacy of ACEIs or ARBs as a result of activated RAS, including AT1R rs5186 [15], AT2R rs11091046 [16], REN rs12750834 [17], ANG rs4762, and ANG rs699 [18], could predispose to a higher risk ofnon-invasive BC. To test this, we conducted a candidate gene association study consisting of two cohorts: one group of patients diagnosed with non-muscle-invasive BC and one group of healthy subjects.

## 2. Materials and Methods

### 2.1. Study Population

Patients of Caucasian origin with a histological diagnosis of non-muscle-invasive urothelial carcinoma of the bladder were prospectively enrolled in this single-center study. Patients harboring tumors with histological variants or upper tract location were excluded. Healthy volunteers including Caucasian men and women >18 years of age were also enrolled and served as the control group. The study was approved by our Institutional Review Board and Ethics Committee and is in accordance with the declaration of Helsinki, as revised in 2013. An informed consent was obtained from each subject before study entry. Clinical and pathological characteristics of patients were recorded, including age, sex, smoking status (active, former, or never smoker), alcohol use (non-drinker, light to moderate drinker, heavy drinker), pathological T stage, grade (low, high). Peripheral blood from healthy subjects and patients was tested for presence of AT1R rs5186 (A1166C), AT2R rs11091046 (C3123A), REN rs12750834 (C5312T), and ANG rs4762 (C620T) and rs699 (T803C) SNPs.

### 2.2. Genomic DNA Extraction

Genomic DNA from peripheral blood samples was extracted with Purelink Genomic DNA (Invitrogen Genomic DNA Mini Kit, Thermo Fisher Scientific, Loughborough, UK), according to the manufacturer’s instructions. DNA was eluted in distilled water and quantified by both agarose gel electrophoresis and absorption spectrophotometry at 260/280 nm.

### 2.3. SNP Genotyping

All five single nucleotide polymorphisms were determined by the polymerase chain reaction-restriction fragment length polymorphism (PCR-RFLP) method. The amplification mixture consisted of 5 μL of 10× reaction buffer, 2.5 mM MgCl_2_, 1.6 mM dNTPs, a 0.1 μM concentration of each primer, 1u Taq DNA Polymerase (ThermoFisher Scientific Inc., Loughborough, UK), and 5 μL of template DNA in a final volume of 50 μL. The amplification conditions are described in Supplementary Table S1. A non-template control (NTC) was included in all PCR reactions. Primer sequences for each SNP are presented in Supplementary Table S2.

### 2.4. Restriction Fraction Length Polymorphism (RFLP) Assay

PCR products for AT1R rs5186, AT2R rs11091046, REN rs12750834, and ANG rs4762 and rs699 were subjected to RFLP analysis using the restriction enzymes DdeI, AluI, NcoI, and SfaNI (10,000 or 2000 U), respectively (New England Biolabs Inc., Hitchin, UK). Then, 10 μL of each PCR product was added in 3 L of digestion buffer along with 15 units of DdeI, AluI, NcoI, or SfaNI, respectively. Distilled water was added to a final volume of 30 μL.

### 2.5. Statistical Analyses

Statistical analysis was performed with IBM SPSS v22 software. Pearson’s chi square was used to evaluate the association of each polymorphism with UCB risk, using the recessive model for the AT1R rs5186 (A1166C), AT2R rs11091046 (C3123A), REN rs12750834 (C5312T), and ANG rs4762 (C620T) and rs699 (T803C) SNPs. The strength of association between SNPs and the development of BC was measured by odds ratios (OR) and relative risks (RR) with 95% confidence intervals (CI). *p* values < 0.05 were considered significant.

## 3. Results

Seventy-three patients with pT1 urothelial BC (66 men, 7 women) and an equal number of healthy subjects (control group) were studied. Molecular evaluation of angiogenic pathways in UC has revealed significant overexpression of key angiogenesis genes, such as VEGF in non-muscle-invasive (89%) compared to muscle-invasive tumors (66%) [19]. In fact, VEGF mRNA expression is up to three-fold higher in superficial compared to muscle-invasive BC [20]. Protein expression of other angiogenesis mediators such as COX2 was also found to be higher in pT1 tumors relative to pTa and pT2 BC [21]. We thus hypothesized that focusing on patients with pT1 disease could provide the highest detection yield for potential genetic associations with RAS gene SNPs. The mean age of our patient cohort was 72 years (Table 1). With regard to environmental carcinogen exposure, most patients were active (*n* = 22) or former (*n* = 35) smokers, whereas only 15 were never smokers. Alcohol consumption was seen in two thirds of patients (*n* = 48), with a minority (*n* = 18) consisting of heavy drinkers (>5 drinks per week) (Table 1). The majority of patients developed high-grade (*n* = 49 or 67%) tumors that were solitary in approximately half of the cases (*n* = 37 or 57%) and multiple in the rest (Table 1). Tumors measuring at least 2 cm or more were found in 28 (46%) patients (Table 1).

The presence of the AT2R rs11091046 SNP, particularly the AA genotype, was associated with a protective effect against developing BC (OR: 0.268 [0.126–0.57], *p* < 0.001) (Table 2). The TT genotype of the REN rs12750834 SNP (OR: 2.8 [1.3–6.05], *p* = 0.008), and to a lesser extent the presence of the T allele (OR: 2.3 [1.2–4.48], *p* = 0.01), conferred a higher risk of BC (Table 2). The highest risk (more than 17-fold) for BC within SNP carriers of the RAS system was associated with the presence of the CC genotype (OR: 17.6 [7.5–41.35], *p* < 0.001) and C allele (OR: 17.7 [8.8–35.9], *p* < 0.01) of the ANG rs699 SNP, respectively (Table 2).

AT1R rs5186 and ANG rs4762 SNPs were not associated with a significant risk compared to healthy subjects.

Upon investigation of a potential relationship between RAS polymorphisms and phenotypic characteristics of urothelial carcinoma in the patient cohort, there were no significant correlations with grade, number, or size of bladder tumors.

## 4. Discussion

In this case-control study, we investigated the potential link between carrying RAS gene SNPs associated with an activated RAS (AT1R rs5186, AT2R rs11091046, REN rs12750834, ANG rs4762, ANG rs699) and the risk of developing non-muscle-invasive BC. Carriers of the CC genotype and C allele of the ANG rs699 SNP were found to have the highest risk, approximately 18-fold higher when compared to non-carriers. The presence of the TT genotype and the T allele of the REN rs12750834 SNP also conferred a higher risk of developing BC, up to 2–3-fold. Conversely, the AA genotype of the AT2R rs11091046 SNP was associated with an approximately 4-fold lower risk of BC.

Our study provides the first evidence for an association between RAS gene SNPs and BC risk. The rs699 C allele encodes the threonine variant, which was until now mainly associated with higher plasma angiotensin levels and a higher risk of hypertension. A weak association with an increased risk of colorectal cancer was previously reported for rs699 in a hospital-based Czech population [22]. Taken together with our results, this could imply an involvement of this SNP in promoting early angiogenesis in BC. Likewise, to a lesser extent, we show that carriers of another hypertension-predisposing SNP, REN rs12750834, can also portend a higher risk of BC, while carriers of the AT2R rs11091046 are protected. From a mechanistic point of view, these findings are in agreement with previous findings of activated AT2R signaling involvement in attenuated tumor growth of various cancers, e.g., lung, pancreatic, or prostate, as well as BC [7,23,24,25].

The role of RAS SNPs in BC angiogenesis was, until recently, underscored. This prospective study is the first to address the clinical relevance of RAS SNPs with respect to BC risk. Further to the addition of RAS gene SNPs in the landscape of genetic factors predisposing to BC, our work could have additional implications for both early and advanced BC in a therapeutic context. This is supported by the fact that breast or colorectal cancer patients carrying RAS genetic variants have differential responses to the anti-angiogenic monoclonal antibody bevacizumab [26]. Although a recent randomized trial of adding bevacizumab to standard cisplatin-based chemotherapy did not show an additional survival benefit in BC [27], perhaps a more focused targeting of a subset of patients carrying more “responsive” RAS gene SNPs could result in clinically significant effects. Additionally, given the active role of immune checkpoint inhibition in the treatment of BC, our study’s findings could pave the way for novel combination therapy approaches, taken together with recent evidence that RAS pharmacologic inhibition with the use of an ACEI or ARB can potentiate the tumoricidal effects of PD-1/PD-L1 inhibitors in patients with metastatic urothelial BC [28]. Thus, knowing the RAS gene SNP status of BC patients could also help target those with the greatest “responsiveness” to both therapies.

The main limitations of this study were the small sample size and the lack of age-matched controls. Additionally, this study focused only on T1 non-muscle-invasive cases, which was however based on the rationale of a more pronounced upregulation of angiogenesis at this stage and thus the highest predicted effect of RAS SNPs.

## 5. Conclusions

In summary, this candidate gene association study supports the clinical utility of the RAS gene SNPs AT2R rs11091046, REN rs12750834, and ANG rs699 in the genetic cancer risk assessment of patients and families with BC. Additional studies are warranted to elucidate the exact composite risk of developing BC, including other genetic [29] and environmental factors (tobacco, alcohol), in order to refine genetic counseling strategies for these patients and their families.

## Figures and Tables

**Table 1:** Clinical and pathological characteristics of the patient cohort.

Characteristic			Patients	
			N	%
Age	Mean ± SD		72.2 ± 10.5	
	Range		48–90	
Gender	Males		66	90.4
	Females		7	9.6
Smoking	Never		15	20.5
	Former		35	48
	Active		22	30
Alcohol use	Never		24	33.3
	Light		30	41.7
	Heavy		18	25
Tumor Grade	Low	1	17	26
	High	2	21	31.8
		3	28	42.2
Foci number	1		37	57
	2–4		9	13.8
	>4		19	29.2
Tumor diameter	<2cm		33	54.1
	≥2cm		28	45.9

**Table 2:** Frequencies of RAS gene SNPs in patients (cases) and healthy subjects (controls).

SNP	Genotype	Patients	%	Healthy Subjects	%	χ^2^ Test	*p* Value	OR (95% CI)	*p* Value
AT1R rs5186	AA	34	46.6	24	36.4				
	AC	36	48.6	38	57.6				
	CC	3	4.1	4	6.1	1.573	0.456		
	A allele	104	71.2	86	65.2				
	C allele	42	28.8	46	34.8	0.926	0.336		
AT2R rs11091046	CC	34	52.3	15	22.7				
	CA	11	16.9	44	66.7				
	AA	20	30.8	7	10.6	33.42	<0.001	0.268 (0.126–0.57)	0.0006
	C allele	79	60.8	77	57				
	A allele	51	39.2	58	43	10.26	0.381	0.86 (0.53–1.4)	0.53
REN rs12750834	CC	2	2.8	3	4.5				
	CT	12	16.9	24	36.4				
	TT	57	80.3	39	59.1	7.324	0.007	2.8 (1.3–6.05)	0.008
	C allele	16	13.7	30	22.7				
	T allele	126	86.3	102	77.3	6.4	0.011	2.3 (1.2–4.48)	0.01
ANG rs4762	CC	55	75.3	44	66.7				
	CT	18	24.7	22	33.3				
	TT	0	0	0	0	1.27	0.259		
	C allele	128	87.7	110	83.3				
	T allele	18	12.3	22	16.7	1.06	0.391		
ANG rs699	TT	0	0	4	6.1				
	TC	11	15.1	46	69.7				
	CC	62	84.9	16	24.2	52.84	<0.001	17.6 (7.5–41.35)	<0.001
	T allele	11	7.5	78	59.1				
	C allele	135	92.5	54	40.9	84.7	<0.001	17.7 (8.8–35.9)	<0.001

## Data Availability

The data presented in this study are available within the article and Supplementary Material.

## Supplementary Materials

The following are available online at https://www.mdpi.com/article/10.3390/curroncol28060396/s1. Table S1: Polymerase chain reaction (PCR) amplification conditions, Table S2: Primer sequences for each SNP.
