# Supplementary material for: Renin-Angiotensin System Single Nucleotide Polymorphisms Are Associated with Bladder Cancer Risk
Source: Curr Oncol. 2021 Nov 15;28(6):4702–8. doi: 10.3390/curroncol28060396 (PMC8628720; doi:10.3390/curroncol28060396)
Supplement: Supplementary file 1 [file curroncol-28-00396-s001.zip › curroncol-1401450-supplementary.pdf]

Current Oncology

Article

Renin-Angiotensin System Single Nucleotide Polymorphisms are Associated with Bladder Cancer Risk

Maria Samara, Maria Papathanassiou, Ioanna Farmakioti, Maria Anagnostou, Maria Satra, Lampros Mitrakas, Dimitrios Anastasiou, Georgios Chasiotis, Agamemnon Christopoulos, Athanasios Anagnostou, Anastasios Christodoulou, Alexandros Daponte, Maria Ioannou, George Koukoulis, Vassilios Tzortzis and Panagiotis J. Vlachostergios

Table S1. PCR Amplification conditions.

Temperature (oC)	Time (mins)	Cycles
94	5	1
94	1	40
56	1	
72	1	
72	10	1
18	hold	

Table S2. Primer sequences for each SNP.

Primer name	Direction (5′-3′)	Sequence
ANG	F	GATGCGCACAAGGTCCTG
ANG	R	CAGGGTGCTGTCCACACTGGCTCGC
AT1R	F	TTGAGGTTGAGTGACATGTTCGA
AT1R	R	CGGTTCAGTCCACATAATGCA
AT2R	F	GGATTCAGATTTCTCTTTGAA
AT2R	R	GCATAGGAGTATGATTTAATC
REN	F	CGTAGTGCCATTTTTAGGAAC
REN	R	AACACCAAAGCAGGCTTAA
